# Long term association of hip fractures by questions of physical health in a cohort of men and women

**DOI:** 10.1371/journal.pone.0283564

**Published:** 2023-03-29

**Authors:** Charlotta Elleby, Pia Skott, Sven-Erik Johansson, Sven Nyrén, Holger Theobald, Helena Salminen

**Affiliations:** 1 Division of Family Medicine and Primary Care, Department of Neurobiology, Care Sciences and Society, Karolinska Institutet, Huddinge, Sweden; 2 Academic Centre for Geriatric Dentistry, Stockholm, Sweden; 3 Public Dental Services, Folktandvården, Stockholm, Sweden; 4 Department of Dental Medicine, Karolinska Institutet, Huddinge, Sweden; 5 Center for Primary Health Care Research, Department of Clinical Sciences, Malmö, Lund University, Lund, Sweden; 6 Department of Molecular Medicine and Surgery, Karolinska Institutet, Solna, Sweden; 7 Academic Primary Care Health Centre, Region Stockholm, Stockholm, Sweden; Ehime University Graduate School of Medicine, JAPAN

## Abstract

We do not know if fracture predicting factors are constant throughout life, if they can be assessed earlier in life, and for how long. The aim was to study the association between questions about health status and mobility and fragility fractures in a cohort during a 35-year follow-up. A cohort of 16,536 men and women in two age groups, 26–45 and 46–65 years old, who answered five questions of their physical health status in postal surveys in 1969–1970. We obtained data on hip fractures from 1970 to the end of 2016. We found most significant results when restricting the follow-up to age 60–85 years, 35 for the younger age group and 20 years for the older. Men of both age groups considered “at risk” according to their answers had a 2.69 (CI 1.85–3.90)– 3.30 (CI 1.51–7.23) increased risk of having a hip fracture during a follow-up. Women in the younger age group had a 2.69 (CI 1.85–3.90) increased risk, but there was no elevated risk for women in the older age group. This study shows that questions/index of physical health status may be associated with hip fractures that occur many years later in life, and that there is a time span when the predictive value of the questions can be used, before other, age-related, factors dominate. Our interpretation of the results is that we are studying the most vulnerable, who have hip fractures relatively early in life, and that hip fractures are so common among older women that the questions in the survey lose their predictive value.

## Introduction

Fragility fractures usually occur late in life, with the median age of hip fractures being 82 years. After a fracture at that age there is often a poor chance of full recuperation, loss of independence and even an elevated risk of mortality [1–3]. It would therefore be desirable to identify high-risk individuals for inset of preventive measures earlier in life. Several methods have been studied, but when in life this identification can take place is not fully known.

Fragility fractures are dependent on bone factors such as bone size and bone mineral density (BMD) and factors concerning falls, such as risk of falling and the protective reactivity when falling. Bone mineral density, measured by Dual-energy x-ray absorptiometry (DXA) at the femoral neck [4], peaks at the age 25–30 years, and declines slowly thereafter. Women usually have a lower peak bone mass than men, and there is a rapid decline after menopause. Fragility fractures are more common late in life due to low BMD, weaker muscles, balance problems, and slower reaction than at a young age. Hence, gender and age are important risk factors. Other examples of known risk factors are corticosteroid medication, low Body Mass Index (BMI), and high alcohol consumption, and the total number of risk factors is found to be more important than the BMD [5].

The most common instrument to estimate fracture risk is the Fracture Risk Assessment Tool (FRAX) [6, 7] which includes risk factors for low BMD, with or without adding the DXA-value. FRAX estimates the ten-year risk of sustaining a major fragility fracture for individuals 40 years or older.

Focusing on the risk of falling rather than the bone density, there are tools testing the physical performance or balance, e.g.”Get-up and Go” [8], “Timed-up-and- go” (TUG) [9] or “One-leg-standing-time” (OLST) [10]. Low muscle strength, or frailty, is also a known risk factor for fragility fractures [11]. Combinations of different testing methods have been suggested, e.g. standing on one foot, grip strength and a question about mobility [12], and narrowing it down to just one simple question of self-rated health on a VAS scale has also been studied and was found to have a fracture predictive value [13].

Many of these studies were done on postmenopausal women or old men, and seldom with long follow-up time. Therefore, it is not known if the fracture predicting factors are constant throughout life, if they can be assessed earlier in life, and for how long. For example, low DXA values was found to predict fragility fractures during a follow-up of up to 25 years in a study of women who were ≥67 years old at study start [14] and in another study of both genders who were ≥45 years of age at study start when followed up to 20 years [15]. Physical performance tests or balance tests have not been studied during a long follow-up time, and very few studies have been done on younger individuals or men. Also, different methods have yielded contradicting results: while the OLST test was found to predict fractures in women 75–80 years old during a follow-up of about 3.6 years [16], the TUG test was not found to predict fractures in adults >75 years during the following year [17]. These varying results point out a lack of knowledge in who can be properly assessed, when and how.

Physical activity in childhood and adolescence are factors known to influence peak bone mass [18]. The physical status may be stable over time and could be used for long term fracture risk assessment. We have access to a large cohort, REBUS, of both genders who were 26–65 years old when they filled in a postal survey in 1969 [19]. Some of these questions concern physical performance, balance, and daily function, reflecting the physical status of the responder. In-patient treated fractures, like hip fractures, have been registered in a national register since around the start of the study, so with this cohort we have a unique possibility to study the hip fracture predictive ability of these questions during a very long follow-up time in a relatively young cohort.

Our aim was to study the hip fracture predictive ability of some questions about daily function and physical ability during a long follow-up time, and if there were age, gender, and follow-up time differences in a cohort with participants 26–65 years old at the start of the study.

## Material and methods

### Cohort

**Fig 1:**
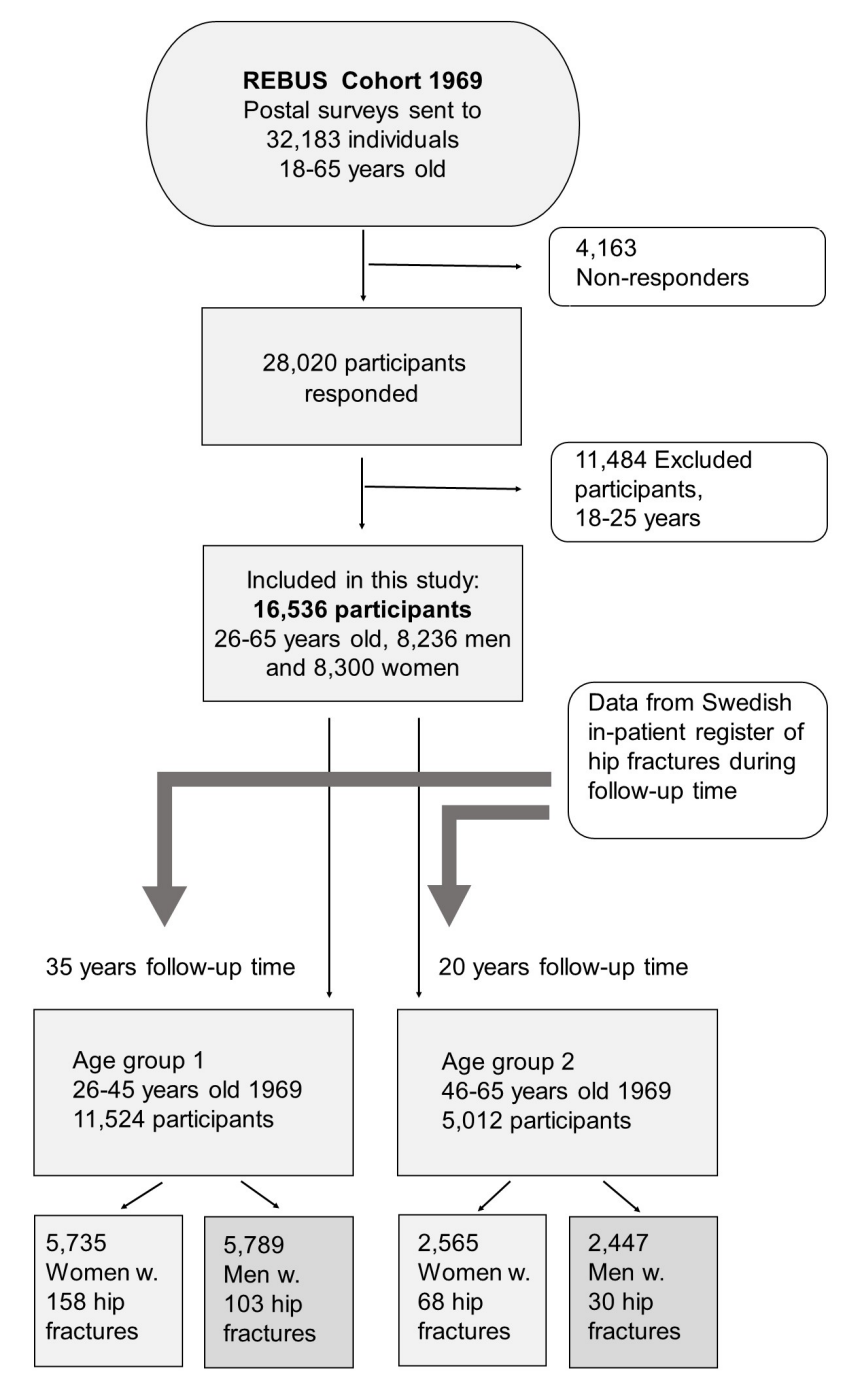
Flow chart of the study population in the present study.

In this cohort study we used five questions from a questionnaire sent to a selected random sample of 32,183, out of the 450 000 inhabitants of the County of Stockholm, in 1969–70 in the REBUS study [19] (Fig 1). The goal of that study was to assess the medical and social situation of the adult population 18–65 years old, and the subsequent need for rehabilitation. Since many disabilities become more frequent with age, proportionally more young individuals were invited to participate in the study. Therefore, 10% of the inhabitants in Stockholm County aged 18–25 years (age group 0), 5% of the inhabitants aged 26–45 years (age group 1), and 3% of the inhabitants aged 46–65 years (age group 2) received the postal survey. The response rate was about 88%. We restricted the participants to age groups 1 and 2 (26–65 years), because we did not consider the youngest age group to be old enough to have enough chronic physical conditions yet. This yielded a total of 18,724 individuals, of which 16,536 answered all five questions, 8,300 women and 8,236 men (Fig 1). The mean and median age for age group 2 was 34 years for both genders, and the mean and median age for age group 3 was 54 years for both genders.

### Questions

The postal surveys that were sent out during four time periods from autumn 1969 to spring 1970 included thirty questions concerning different social, psychological, and medical topics. We used the five questions concerning physical performance, balance, and daily function that could be related to risk of falling.

The questions included in our study were:

Q1: Do you have difficulties in doing your daily activities because of poor physical health?

Q2: Do you feel sick and unwell?

Q3: Are you troubled by dizziness?

Q4: Do you have difficulties climbing the stairs?

Q5: Do you have difficulties walking indoors?

There were three possible tick-box answers to each question: “often”, “sometimes” or “never”.

Participants who had answered “often” or “sometimes” to the individual questions were labelled “at risk”. An index was constructed using all five questions, where answering “often” to at least one of them labelled the participants “at risk” (Table 1). In age group 1 (26–45 years) less than 20% were “at risk” according to all questions except question 2 (Do you feel sick and unwell?) to which 51.8% of the women and 39.3% of the men were considered “at risk”. In age group 2 (46–65 years) more participants were labelled”at risk” than in age group 1 for all five questions, which was expected as physical ability generally is reduced with age.

**Table 1:** Baseline characteristics for our sample of the REBUS cohort 1969–70.

Variable	Age group 1, 26–45 years			Age group 2, 46–65 years		
	Follow-up 35 years			Follow-up 20 years		
	**Women**	**Men**	**Total**	**Women**	**Men**	**Total**
Gender, n	5,735	5,789	11,524	2,565	2,447	5,012
Mortality at end of study n, (%)	689 (12.0%)	1,168 (20.2%)	1,857 (16.1%)	493 (19.2%)	885 (36.2%)	1,378 (27.5%)
Median age of death, years (IQR)	74 (65–81)	72 (62–79)	73 (63–80)	84 (76–90)	78 (70–86)	81 (73–88)
Hip fractures n, (%)	158 (2.8%)	103 (1.8%)	261 (2.3%)	68 (2.7%)	30 (1.2%)	98 (2.0%)
Q1. Do you have difficulties in doing your daily activities because of poor physical health? Risk* %, (n)	18.7 (1075)	14.7 (849)	16.7 (1,924)	34.6 (888)	29.1 (713)	32.0 (1,601)
Q2. Do you feel sick and unwell? Risk* %, (n)	51.8 (2,971)	39.3 (2,274)	45.5 (5,245)	61.4 (1,574)	48.6 (1,188)	55.1 (2,762)
Q3. Are you troubled by dizziness? Risk* % (n)	28.3 (1,622)	12.3 (713)	20.3 (2,335)	38.1 (976)	21.6 (528)	30.0 (1,504)
Q4. Do you have difficulties climbing the stairs? Risk* % (n)	11.6 (663)	6.4 (370)	9.0 (1,033)	33.7 (863)	21.9 (535)	27.9 (1,398)
Q 5. Do you have difficulties walking indoors? Risk* % (n)	4.1 (234)	3.2 (183)	3.6 (417)	10.2 (262)	6.8 (167)	8.6 (429)
Index (all five questions) Risk**, % (n)	5.9 (337)	3.8 (219)	4.8 (556)	15.4 (394)	11.7 (287)	13.6 (681)

* Q1-Q5risk is having answered “often” or “sometimes” to questions 1–5.

** Index risk is having answered any of the questions 1–5 with “often”.

### Fracture data

Data on hospital admissions due to hip fractures from 1^st^ of January 1970 to the end of the study, 31^st^ of December 2016, in total 47 years follow-up, were acquired from the National Patient Register, kept by the Swedish National Board of Health and Welfare. This registry started in 1969 and was gradually introduced until full coverage of in-patients in the Stockholm area from 1972 and validated by Ludvigsson et al. [20]. We used hip fractures as an example of fragility fractures because they are the only ones that are seldom undetected and always require hospitalization. The data of hip fractures from the National Patient Register used, were codes 820, 821, 8200, 8201, 8210, 8211 according to ICD8 (1969-01-01–1986-12-31); codes 820, 820A, 820B, 820C, 820D, 820W, 820X, 821, 821A, 821B according to ICD9 (1987-01-0 1–1996-12-31); and codes S720*, S721*, S722*, S723*, S72 according to ICD10 (1997-01-01 -). depending on when they occurred. Our definition of “hip fracture” includes fractures of the femoral neck and peri- and sub-trochanteric fractures. The different ICD systems do not match completely, and sometimes the data was incomplete from the register, so when in doubt of the exact location of the fracture we chose to include rather than to omit in order not to miss any events.

We did not have access to the codes indicating what kind of trauma that had caused the fracture for all events of hospitalization as the registers are not complete, so we were not sure all hip fractures were due to low trauma. However, when we checked this in an earlier study of a sample of the same cohort using the ICD-10 system that is more complete, we found that of 153 hospitalizations due to hip fracture, 99 were due to a fall in the same plane, and only 9 were due to a fall from a higher level, and 45 had no registered cause [21]. Others have found that excluding high energy trauma may underestimate the prevalence of fragility fractures [22] and we are advised not to make the distinction between fractures resulting from high or low energy trauma by Cummings and Eastell [23]. We therefore decided to consider all hip fractures to be due to low trauma. If one individual had several events of hospitalization due to hip fracture during the follow-up time, we only included the first because we could not separate events due to new fractures from events due to complications of a previous hip fracture.

The date of death for those individuals who were deceased at the end of the study was acquired from The Swedish National Board of Health and Welfare, who has kept records of mortality data in the Cause of Death Register since 1952. We did not use data of hip fractures from the cause of death register as this added very few events when we checked.

### Statistical analyses

The statistical analyses were performed using Stata/IC 14.2 for Windows (StataCorp. 2015. Stata Statistical Software: Release 14. College Station, TX: StataCorp LP). The significance level was set at p-values ≤0.05. Comparative statistics, with significance testing of gender differences of variables, was performed using the Chi-2 test. A Poisson regression model, adjusted for gender and time-varying age, was used, calculating the incidence rate ratio (IRR) with 95% confidence interval (CI), and the first hip fracture as failure. Individuals who survived the study without fracture were censored at the end of the study. Age-adjusted incidence rates of hip fractures for men and women by Question/Index were estimated by using Poisson regression and as post-estimation command we used margins.

Since Cox model requires proportionality over time, we tested the Hazard Ratio (HR) and found lack of proportionality over time. We therefore chose to use the Poisson model, using time split at failures, resulting in such small time bands that we could assume proportionality within the time bands. We analyzed women and men together, but when there was an interaction term between gender and item (question or index), we included a gender*item(question or index) interaction term. P-values for Goodness of Fit-tests were all ≥0.05, thus satisfactory fit.

We also used a parametric model including competing risk according to to Lambert [24] for analysis. This was done after using the stcrreg command in Stata.

### Ethical approval and consent to participate

This study and the original REBUS study were performed in accordance with the ethical standards of the institutional and/or national research committee and with the 1964 Helsinki declaration and its later amendments or comparable ethical standards. The participants gave their informed consent at the time of the original REBUS study. This study was approved by the Regional Ethical Review Board Stockholm June 22, 2016 (Registration number 2016/902-31/2). The first ethical committee, which was only advisory, was formed at Karolinska Institutet, Stockholm in 1966. No formal ethical application was needed for the original study, but the committee was aware of the study and did not object. Follow-up studies have been approved, before 2004 by the local ethics committee at Karolinska, and after 2004 by the Regional Ethics Review Board Stockholm. For follow up studies in 1996 the ethics committee required that the participants were informed that their data was to be used for this and future studies through advertisements in the local papers, which was satisfactory done. The ethics committee, and later the Regional Ethical Review Board, has since then considered the participants to have given their consent for future studies.

We have access to the digitalized data of participants, including social security numbers, with the answers of the postal surveys. These were sent to the Swedish National Board of Health and Welfare for addition of fracture data and returned without any identification numbers, only date of birth and gender.

## Results

There were 16,536 individuals in the cohort, divided into two age-groups. The follow-up period started January 1^st^, 1970. We found that the fracture predictive value of the questions was most significant when restricting the follow-up time to 35 years in the younger age group, thereby followed to 61–80 years of age. For the older age group, the same was true for a follow-up time of 20 years, up to 66–85 years of age. During the 35-year follow-up of group 2, 261 individuals (2.3%) had at least one hip fracture (Table 1), 16% had died without having a hip fracture, and 82% survived the study without hip fracture. During the 20-year follow-up of group 2, 98 individuals (2.0%) had at least one hip fracture, 27% were deceased without having a hip-fracture, and 71% survived the study without hip fracture (Table 1). The age-adjusted incidence rate for hip fractures in the two age groups for women and men for each question are shown in Table 2.

**Table 2:** Age-adjusted incidence rates per 100,000 person years with 95% confidence intervals (95% CI) for hip fractures by sex.

Item		Age at baseline		Age at baseline	
		26–45 y (n = 11,524, 261 failures)		46–65 y (n = 5,012, 98 failures)	
1 = risk*		Women (95% CI)	Men (95% CI)	Women (95% CI)	Men (95% CI)
0 = no risk					
Q1. Do you have difficulties in doing your daily activities because of poor physical health?	1*	114 (85–143)	79 (55–102)	192 (131–254)	101 (58–144)
	0	77 (63–90)	53 (42–64)	126 (88–164)	66 (40–92)
Q2. Do you feel sick and unwell?	1*	96 (79–114)	68 (52–83)	174 (127–221)	95 (57–132)
	0	71 (56–86)	50 (38–61)	113 (69–156)	61 (34–88)
Q3. Are you troubled by dizziness?	1*	103 (79–127)	73 (51–95)	181 (123–239)	98 (53–142)
	0	77 (62–91)	54 (44–65)	131 (91–171)	71 (44–98)
Q4. Do you have difficulties climbing the stairs?	1*	146 (104–187)	104 (69–138)	182 (123–241)	98 (53–143)
	0	75 (62–87)	53 (43–64)	130 (90–170)	70 (43–97)
Q5. Do you have difficulties walking indoors?	1*	167 (96–238)	114 (63–165)	163 (65–261)	84 (27–141)
	0	80 (67–93)	55 (44–65)	149 (111–186)	77 (49–104)
Index (all five questions)	1**	189 (122–256)	132 (81–184)	202 (113–291)	105 (49–162)
	0	77 (64–90)	54 (43–64)	140 (103–176)	73 (46–100)

*Q1-Q5 risk (= 1) is having answered “often” or “sometimes” to the questions 1–5.

**Index risk (= 1) is having answered “often” to any of the questions 1–5

In age group 1, the analyses showed that, for both genders, the Incidence Rate Ratio (IRR) of hip fracture was elevated for those who were “at risk” for all five questions; IRR ranged from 1.37 (CI 1.04–1.81) to 3.78 (CI 2.36–6.06) (Table 3).

**Table 3:** Poisson regression of hip fractures, adjusted for gender and time-varying age, with risk according to the five questions.

	Age group 1			Age group 2		
	26–45 years at baseline			46–65 years at baseline		
Questions	IRR	95% CI	p	IRR	95% CI	p
Q1. Do you have difficulties in doing your daily activities because of poor physical health?	1.55	1.17–2.06	0.002	1.63	1.09–2.43	0.017
Q2. Do you feel sick and unwell?	1.39	1.09–1.78	0.008	1.64	1.07–2.51	0.023
Q3. Are you troubled by dizziness?	1.37	1.04–1.81	0.025	1.43	0.95–2.14	0.088
Q4. Do you have difficulties climbing the stairs?	3.78 (Men)	2.36–6.07	<0.001	1.48	0.98–2.24	0.062
	1.50 (Women)	1.00–2.25	0.049			
Q5. Do you have difficulties walking indoors?	2.21	1.42–3.42	<0.001	1.16	0.62–2.16	0.65
Index including all questions	2.69	1.85–3.90	<0.001	3.30 (Men)	1.51–7.23	0.003
				1.10 (Women)	0.59–2.05	0.77

Q1-Q5 risk (= 1) is having answered “often” or “sometimes” to the questions 1–5.

Index risk (= 1) is having answered “often” to any of the questions 1–5.

When there was a significant gender*item (question or index) interaction term, men and women were analyzed separately (applied for Q4 in age group 1 and Index in age group 2).

For question 4 (Q4) (“Do you have difficulties climbing the stairs?”) we had to analyze the genders separately because of the interaction term between gender and Q4. For the index (having answered any of the questions 1–5 with “often”) IRR was 2.69 (1.85–3.90) in age group 1. In age group 2 only Q1 (“Do you have difficulties in doing your daily activities because of poor physical health?” and Q2 “Do you feel sick and unwell?”) resulted in elevated IRR for both genders, 1.63 (CI 1.09–2.43) and 1.64 (CI 1.07–2.51) respectively. For age group 2, the index had an interaction term and genders had to be analyzed separately, resulting in an elevated IRR of 3.3 (CI 1.51–7.23) for men, but no significant change for women 1.10 (CI 0.59–2.05) for women.

In our study 16–28% of the participants died during the follow up time and were thereby exposed to the competing risk of death. Using the Competing risk model with Stata proposed by Paul C. Lambert [24] we acquired similar results as Poisson.

**Fig 2:**
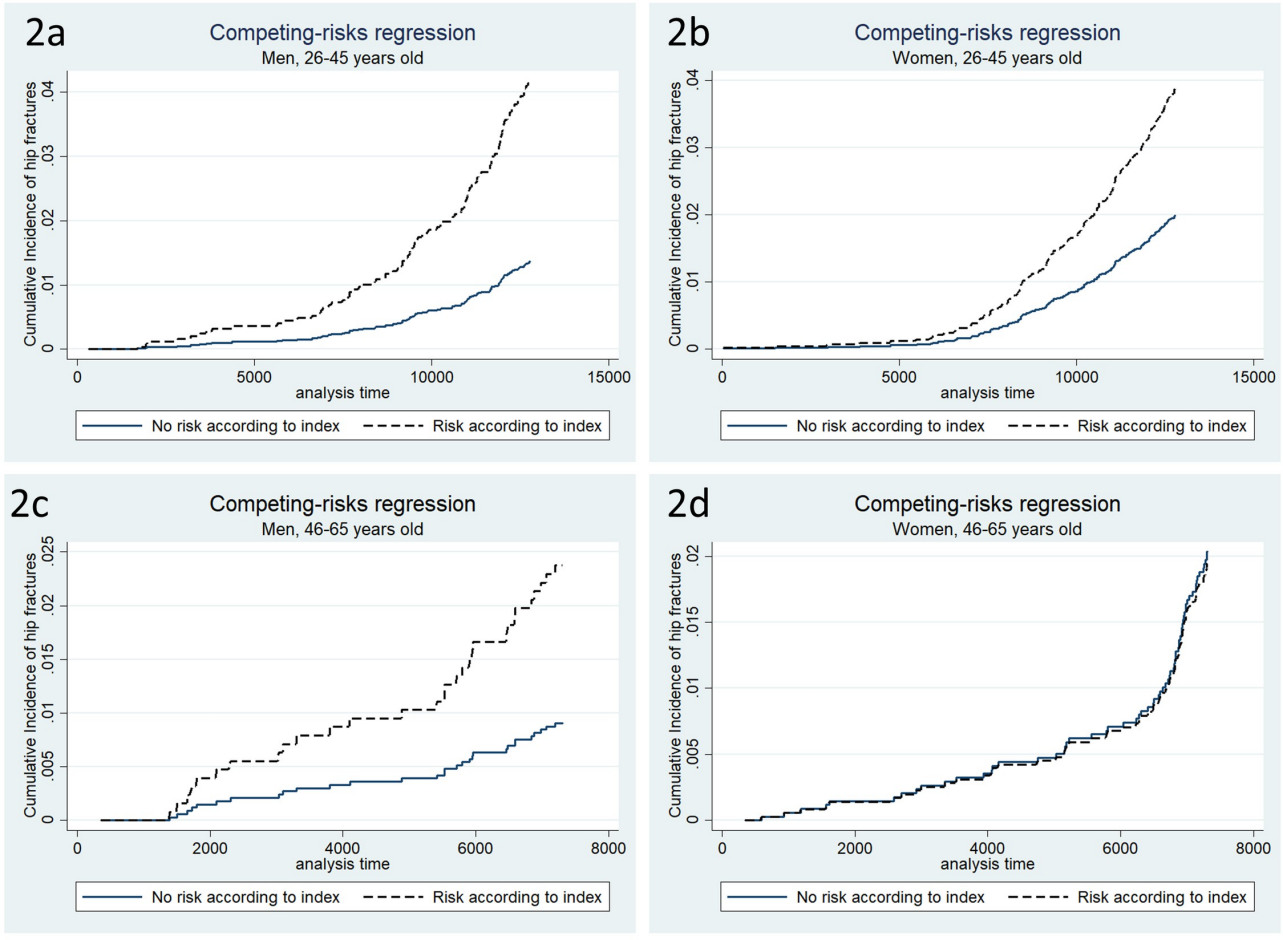
Cumulative incidence function of hip fractures. Cumulative Incidence of hip fractures by gender and age group after using the stcrreg command in Stata for Competing risks regression according to Fine & Gray.

Fig 2A and 2B show that there is a difference in the cumulative incidence function (CIF) for hip fractures between “at risk” and “not at risk” according to the index in age group 1, regardless of gender. Fig 2C and 2D show the CIF for hip fractures “at risk” and “not at risk” according to the index in age group 2 where we had to analyse the genders separately because of the interaction. There is a distinct difference in CIF for men between those who were “at risk” or “not at risk”, but no difference for women.

## Discussion

Using an index indicating “at risk” according to five questions in a postal survey from 1969–70, we found an approximately three times higher risk of sustaining a hip fracture during the 20- or 35-year follow-up for men in both age groups. Women in the younger age group had also an approximately three times higher risk of sustaining a hip fracture, but there was no elevated risk for women in the older age group.

When testing when and how long the fracture predictive value of the questions was significant, we found that for both age groups, they had to be followed until they reached an age of 60–85 years. A possible explanation for this is that since hip fractures usually occur late in life (80+), the participants were too young before this age to have hip fractures which resulted in too few hip fractures for our statistical analysis. However, longer follow-up time than 20 or 35 years did not yield significant prediction of fractures, which implicates, according to our interpretation, that other, age-related, factors become more important, such as balance, reaction time and frailty. Also, the average life expectancy rose from 1970 to 2004 from 72.2 years for men and 77.1 years for women, to 78.4 years for men and 82.7 years for women [25]. This means that many of the participants died before they reached the age where hip fractures are most common today. Our conclusion is therefore that we may be looking at the most “vulnerable” in our study, those who have a hip fracture relatively early in life.

We found that older women with an augmented risk of sustaining a hip fracture cannot be identified with our method. Our interpretation of this is that, since women have a rapid decline in BMD after menopause, this has a higher impact on their fracture risk than the questions in our study. Fragility fractures in women are so common that it will eventually happen also to women with good physical health and balance when they are older. Women who are younger but already have a bad physical health may be overrepresented with hip fractures later in life, hence the significantly elevated IRR.

Did the introduction of medication for osteoporosis influence the results in our study? Medication for osteoporosis became available in Sweden in 1995, when bisphosphonates were registered for commercial use. This could potentially have influenced the results for the younger age group during the last ten year of their follow-up until 2005. The use of bisphosphonates rose during this period, but only 0,38% of the population had a prescription for bisphosphonates in the County of Stockholm in 2004 [26]. The treatment gap was considerable, in 2010 it was calculated to be 60–70% in Sweden [27]. Because of poor compliance and persistence, only about 25% still took their bisphosphonate medication after 1–2 years [28, 29]. Swedish studies show similar results [30]. The effect of bisphosphonates on fractures incidence is only about 40%, additionally minimizing the effect of medication to the results in our study [31]. We therefore conclude that medication for osteoporosis does not have a significant effect on the results of our study.

The strength of this study is the large study group (16,536 participants) and the possibility of a long follow-up time, up to 47 years. The response rate was 88%, which is very high. This means that the probability of a representative sample of the population should be high. We have also analyzed the data with three different methods that all gave the same results. We chose to present the results from the Poisson analysis in this study, because lack of proportionality over time made Cox unsuitable. Using competing risk according to Fine and Gray [32] is often recommended but has been criticized [33] and we chose not to present the detailed data here.

The limitation of the study is that the study start was over 50 years ago, and the lower life expectancy at this time means that many of the older participants in the study may not have reached the age where hip fractures are most common today. Not including the trauma type could be a limitation, but as previously described, we tested this in a smaller sample of the cohort and found that most fractures were due to low energy trauma. Combined with findings of other studies that exclusion of high trauma fractures underestimates fragility fractures [22] and that high trauma fractures also contributes to fragility fractures [34] we do not find this lack of trauma type a problem.

To fill a knowledge gap, we have studied some risk factors of fragility fractures during a long follow-up time, to identify when, and for how long they have a predictive value. In our study the risk factors are questions about physical health and mobility, and we have analysed their association to hip fractures during a follow-up time up to 47 years. For younger individuals (26–45 years old when they answered the survey) we found a predictive value when we included hip fractures during the follow-up time until they reached an age of about 60–85 years. For older individuals (46–65 years) the same was true for men, but not for women, who have more hip fractures at an earlier age than men. This indicates that there is a certain period during mid-life when questions about health can be used to identify individuals with a high risk of fragility fractures. However, this effect is washed out as they get older and other, age-dependent, factors dominate, and this occurs earlier for women than for men. These findings could be used when considering the use of risk-predictive instruments and planning preventive measures, such as medication, and is of such interest that further studies are motivated.

## Conclusion

This study shows that questions of physical health may be associated with hip fractures that occur many years later in life, and that there is a time span when the predictive value of the questions can be used, before other, age-related, factors dominate.

## Supporting information

S1 Dataset (XLSX)
